# Stabilization Strategies for Unstable Dynamics

**DOI:** 10.1371/journal.pone.0030301

**Published:** 2012-01-18

**Authors:** Devjani J. Saha, Pietro Morasso

**Affiliations:** 1 Department of Biomedical Engineering, Northwestern University, Chicago, Illinois, United States of America; 2 Robotics, Brain and Cognitive Sciences (RBCS) Department, Istituto Italiano di Tecnologia (IIT), Genoa, Italy; 3 Dipartimento di Informatica, Sistemistica e Telematica (DIST), University of Genoa, Genoa, Italy; The University of Western Ontario, Canada

## Abstract

**Background:**

When humans are faced with an unstable task, two different stabilization mechanisms are possible: a high-stiffness strategy, based on the inherent elastic properties of muscles/tools/manipulated objects, or a low-stiffness strategy, based on an explicit positional feedback mechanism. Specific constraints related to the dynamics of the task and/or the neuromuscular system often force people to adopt one of these two strategies.

**Methodology/Findings:**

This experiment was designed such that subjects could achieve stability using either strategy, with a marked difference in terms of effort and control requirements between the two strategies. The task was to balance a virtual mass in an unstable environment via two elastic linkages that connected the mass to each hand. The dynamics of the mass under the influence of the unstable force field and the forces applied through the linkages were simulated using a bimanual, planar robot. The two linkages were non-linear, with a stiffness that increased with the amount of stretch. The mass could be stabilized by stretching the linkages to achieve a stiffness that was greater than the instability coefficient of the unstable field (high-stiffness), or by balancing the mass with sequences of small force impulses (low-stiffness). The results showed that 62% of the subjects quickly adopted the high-stiffness strategy, with stiffness ellipses that were aligned along the direction of instability. The remaining subjects applied the low-stiffness strategy, with no clear preference for the orientation of the stiffness ellipse.

**Conclusions:**

The choice of a strategy was based on the bimanual coordination of the hands: high-stiffness subjects achieved stability quickly by separating the hands to stretch the linkages, while the low-stiffness subjects kept the hands close together and took longer to achieve stability but with lower effort. We suggest that the existence of multiple solutions leads to different types of skilled behavior in unstable environments.

## Introduction

In recent years, the neural control of unstable tasks has been the topic of many studies [1], [2], [3], [4], [5], [6]. In general, instability is induced through a divergent force field that pushes the state of the system away from an unstable equilibrium. The divergent field produces a “toppling” force or torque that grows with the distance of the current state of the system from the equilibrium state. An inverted pendulum is a typical example of an unstable system, with many variations that are either ecologically inspired (e.g. upright bipedal standing or balancing a rod, where the divergent field is due to gravity) or artificially/virtually produced (e.g. balancing a virtual mass in a robot-generated force field). When humans are faced with such tasks, in principle two different stabilization mechanisms are possible:

1. A high-stiffness strategy (SSS), based on the elastic properties of the body/environment system, which induces a convergent, restoring force field. This field can successfully compensate for the source of instability if its stiffness (or its rate of growth) is greater than the rate of growth of the divergent, toppling field. Thus, in order to apply the SSS and overcome the dynamic effects of the unstable field a critical value of stiffness (*K_c_*) must be obtained. We may also consider stiffness as an implicit positional feedback, which has a nearly instantaneous response time. The overall stiffness that interacts with the environment consists of stiffness from muscles, tendons, tools, and manipulated objects. However, if elastic elements are connected in series the most compliant element dominates the overall stiffness of the system.
2. A low-stiffness strategy (PSS) is based on explicit positional feedback from different sensory channels (e.g. proprioception and vision). This strategy is necessary if the overall intrinsic stiffness is weak or totally absent, as in the case of the pole-balancing problem. The PSS can be implemented by means of a servomechanism, which is closed-loop in nature and involves continuous time control with high-gains [7], [8]. However, this is an unfeasible solution, due to long delays in the feedback loop which itself becomes a source of instability. A more robust solution is to close the loop intermittently, by injecting force impulses in the system through predictive control or based on sensory input [1], [9], [10], [11].

If the divergent field is directly applied to the body, then the relevant elastic elements are muscles and tendons of the operating joints. For example, in the experiments described by Burdet et al. [2] the field is applied to the hand and interacts with elbow and shoulder muscles. The stiffness of these muscles is always smaller than the stiffness of the tendons. This allows the subjects to modulate the overall stiffness of the hand by means of muscle coactivation and moreover learn coactivation patterns that are optimal for the task. This is a typical condition for applying the SSS. In contrast, for upright standing subjects apply a PSS strategy. The reason is that the Achilles tendon at the ankle, the joint most responsible for balance during standing, is more compliant than the plantar flexors of the ankle [12]. Therefore, the ankle stiffness is dominated by the stiffness of the tendons rather than the co-contraction of muscles. Even if the SSS were to be adopted, it would have little effect on the overall stiffness of the ankle.

In other cases, the divergent field is not applied directly to the body but indirectly, via an elastic tool. Consider, for example, a hinged bar which is kept upright by means of a hand-held elastic linkage. If the linkage is sufficiently stiff, then a SSS can be used to overcome the rate of growth of the toppling torque. However, somewhat paradoxically, people can also learn to balance an unstable system with linkages that have stiffness lower than the critical level [4]. Because the overall stiffness is dominated by the uncontrollable stiffness of the soft linkage, subjects can not implement the SSS. Instead they can prevent the bar from falling down by applying force impulses via a hand-held elastic element (PSS strategy) that is connected to the bar. Here, the critical factor is not stiffness but the time constant of the incipient fall (*T_fall_*) in relation to the response time of the controller (*T_c_*). If *T_fall_*<*T_c_*, then the observer does not have enough time to provide the correction bursts necessary for stabilization and the task becomes impossible.

In general, we can classify the experimental conditions, which characterize the feasibility of the stabilization strategies according to Table 1, where *K_c_* is the critical stiffness value determined by the source of instability and *K_min_* to *K_max_* defines the range of stiffness values of the limb/manipulated object that can be achieved.

**Table 1:** Stabilization Strategy.

Stabilization Strategy Table	*Tfall<Tc*	*Tfall>Tc*
*Kmin<Kc<Kmax*	C1**SSS**	C2**both**
*Kmax<Kc*	C3**impossible**	C4**PSS**

Feasibility of a stabilization strategy based on 1) the relationship between the response time (*T_c_*) and the time constant of the unstable task (*T_fall_*), and 2) the relationship between the critical stiffness (*K_c_*) and the stiffness of the controller (which ranges from *K_min_* to *K_max_*).

Four paradigmatic conditions can be identified, which correspond to areas of feasibility of the two stabilization strategies. For example, the study by Burdet et al. [2] can be classified as condition C1, whereas pole balancing or the study by Lakie et al. [4] can be classified as condition C4. However to our knowledge, little attention has been given to condition C2 where both stabilization strategies are possible. The C2 condition allows us to investigate a variety of problems related to selection of stabilization strategies, strategy-switching, and generalization.

In order to address whether subjects are biased toward a particular stabilization strategy when presented with an unknown dynamical system we designed an unstable task in which both stabilization strategies were feasible, although with strongly different outcomes in terms of effort and stability. The experimental task used a bimanual, planar robot to simulate two virtual elastic linkages connected on one end to the robot handles and on the other end to a virtual mass. The virtual mass was under the action of a saddle-like force field. The two linkages were non-linear, such that the stiffness of each linkage increased with the amount of stretch. By applying forces to the two robot handles the overall stiffness ellipse of the system could be modulated. Specifically, stiffness orientation depends on the orientation of the two hands relative to the mass and its size depends on the amount of stretch at the two linkages. The goal of the task was to stabilize the virtual mass in various target areas of the workspace. Due to the presence of a saddle-type force field, which is characterized by a divergent component along the mediolateral direction, the task is unstable. The parameters of the task were calculated such that both stabilization strategies were possible, i.e. the falling time constant was larger than the critical level and the stiffness that could be achieved were greater than the critical stiffness of the divergent field. Moreover, the geometrical parameters and the size of the workspace were chosen such that the task was fatiguing but doable.

Although the experimental setup was unique, certain components of the task were inspired by neurophysiological processes. For example, the idea of stretching two opposing springs to increase stiffness is analogous to the concept of coactivation of antagonistic muscle groups to increase joint stiffness [13], which consequently leads to an increase in hand stiffness [14]. Moreover, stiffness modulation of the hand has been attributed to selective co-contraction of different elbow and shoulder muscles [15], [16]. However, as indicated by Perreault et al. [17], voluntary changes in stiffness orientation of the hand is limited in isometric conditions and largely constrained by the force applied by the hand. In contrast, changes in posture of the arm can dramatically change the orientation of hand stiffness [18], [19]. In order to reflect the voluntary regulation of stiffness observed in human behavior, we designed a setup that allowed subjects to independently modulate not only the magnitude but also the orientation of the stiffness associated with the spring mass system.

For simplicity, we limited our attention to task dynamics rather than to the underlying patterns of muscle activation [20], which itself is an issue. There is ample literature that suggests that tool use involves modifications at the central level in which tools become extensions of the ‘Body Schema’ [21]. This supports the idea of focusing on task dynamics rather than muscle dynamics, at least for the preliminary study of the motor control of a complex, unstable task like the one considered in this paper. Our work is also related to the recent paper by Ganesh et al [22], which investigated the behavior of subjects in a task that also allows for multiple solutions. Their task involved guided exploration of the solution space in order to assess whether subjects adopted a suboptimal solution after exposure to a global optimum. In the cited study, subjects had implicit knowledge of both the optimal and suboptimal trajectories. However, in our experiment subjects were left free to explore the solution space without initial knowledge of two different strategies. Although the task was designed such that subjects were exposed to both solutions, it was not immediately obvious which strategy naive subjects would finally adopt. In fact, the goal of this study was to provide preliminary knowledge on the issue of strategy selection in unstable tasks. Our study characterizes the behavior of naïve subjects in the initial phase of learning. Additional studies will be needed for modeling the behavior of expert users and the mechanism of strategy switching in demanding situations.

## Materials and Methods

### Ethics Statement

The research conforms to the ethical standards laid down in the 1964 Declaration of Helsinki that protects research subjects. Each subject signed a consent form that conforms to these guidelines. The research obeys with the protocol “Studio di paradigmi di controllo motorio e adattamento a campi di forza nell'arto superiore mediante utilizzo di interfacce robotiche interattive” (Study of paradigms of motor control and adaptation to force fields in the upper limb by means of interactive robotic interfaces) approved by the “Comitato Etico” (Ethical Committee) of “ASL 3 Genovese” (the Local Health Authority) which is legally competent for approving experiments involving human subjects.

### Subjects

Thirteen healthy, right-handed adults (age = 27±3.7 y, four females) participated in the experiment (Table 2). Hand preference was evaluated by means of the Edinburgh Handedness questionnaire [23]. One of the issues of the experiment was to determine whether physical parameters, which are indicative of ‘arm strength’, were relevant factors in strategy selection. For this purpose we used three parameters: 1) body weight, 2) body mass index (BMI calculated as the ratio between weight and squared height), and 3) maximum grip force (measured with a hydraulic hand dynamometer by Baseline Evaluation Instruments). The parameter values for each subject are reported in Table 2.

**Table 2:** Subject Data.

Subject	Gender	Height (cm)	Weight (kg)	BMI (kg/m^2^)	Grip Force (N)	Strategy
1	M	167	63	23	412	SSS
2	M	170	65	22	432	SSS
3	F	157	54	22	235	PSS
4	M	181	75	23	618	SSS
5	F	170	60	21	334	PSS
6	M	170	59	20	353	PSS
7	F	173	60	20	284	PSS
8	M	184	80	24	343	SSS
9	M	180	81	25	432	SSS
10	F	168	56	20	235	SSS
11	M	174	63	21	530	PSS
12	M	182	88	27	402	SSS
13	M	169	59	21	412	SSS

Anthropometric data from each subject. The last two columns also include the maximum value of grip force and the stabilization strategy applied during the last two target sets, respectively.

### Apparatus

**Figure 1:**
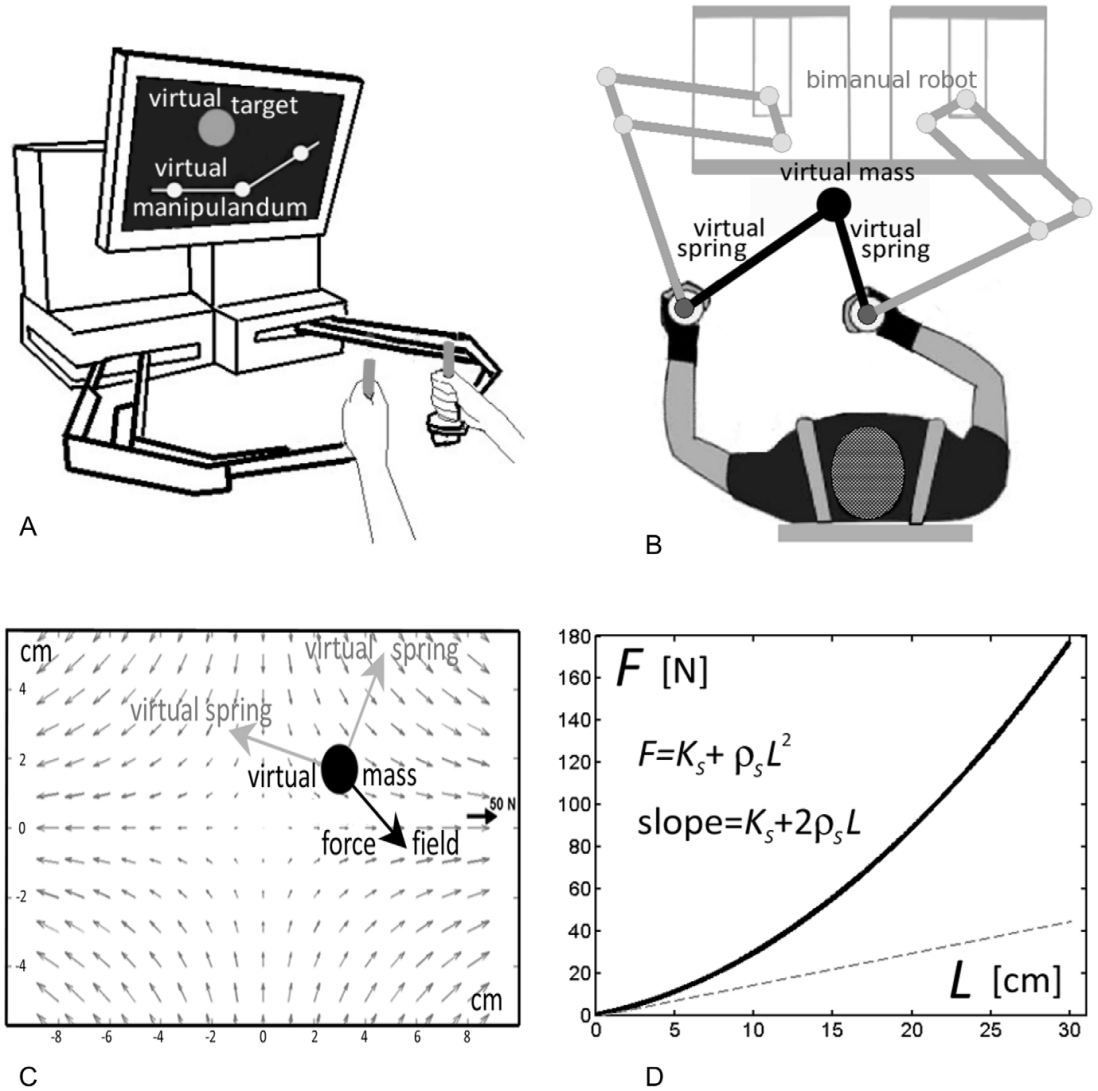
Main features of the experimental setup. A: Bimanual haptic robot (BdF2, Celin srl, La Spezia, Italy). B: Virtual Underactuated Bimanual Manipulandum (VUBM) simulated by BdF2. C: force field applied to the virtual mass. D: length-tension curve of a non-linear virtual spring.

The experiments are based on a virtual, underactuated, bimanual manipulandum (VUBM), which is simulated by means of a bimanual haptic robot (BdF2, Celin srl, La Spezia, Italy, a direct evolution of the uni-manual robot manipulandum Braccio di Ferro [24]). Each robot has a large planar workspace (80×40 cm ellipse) and a rigid structure with two direct-drive, brushless motors, and low intrinsic mechanical impedance. These features allow direct estimation of hand forces from the commanded currents to the motors and the Jacobian matrices of the robots. Each robot can measure the trajectory of the hand with high-resolution (0.1 mm) and is capable of applying forces at the corresponding handle. The control architecture is based on the real-time operating system RT-Lab® and includes three nested control loops: 1) an inner 16 kHz current loop, 2) an intermediate 1 kHz impedance control loop, and 3) an outer 100 Hz loop for visual display and data storage. The two identical planar robots are mounted in a mirror configuration on the same rigid frame, which allows independent regulation of vertical and horizontal position (Figure 1). They are positioned horizontally as close as possible (distance between the axes of the motors 38.5 cm), in order to maximize the overlap between the corresponding workspaces. The vertical position of the robot linked to the right hand was adjusted such that the right arm was approximately horizontal. The other robot was slightly shifted downward in order to avoid interference between the two arms. The vertical distance between the two hands (second metacarpal joint) was 18 cm. The positions of the two handles were calibrated with respect to a common reference frame, which was used for all the relevant variables of the experiments.

VUBM (Figure 1) consists of two virtual elastic linkages attached, on one side, to a virtual mass M of 15 kg and, on the other, to the two handles grasped by the subjects. The two elastic linkages are nonlinear and are characterized by the sum of two length-tension curves, one that increases quadratically and another that increases linearly with the length of each linkage:
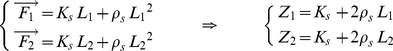
(1)
*L_1_* and *L_2_* are the lengths of the two virtual springs; 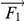
and 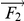 are force vectors that are applied by the two linkages to the handles; and Z_1_ and Z_2_ are the corresponding stiffness values. The forces are oriented along a straight-line connecting the mass to the handle. In addition to the forces applied by the two handles, the virtual mass-load is also under the action of an unstable, saddle-like force field (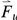):
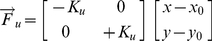
(2)where [*x,y*] identifies the position of mass-load and [*x_0_,y_0_*] is the origin of the force field, which is located in the center of the common workspace of the two robots. The unstable manifold of the field is aligned mediolaterally, or along the x-axis of the workspace, while the stable manifold is aligned anterioposteriorly along the y-axis of the workspace.

The subjects must learn to stabilize the mass in different parts of the workspace by acting on the positions of the two handles that indirectly affect the lengths of the two linkages and the corresponding forces. The VUBM is underactuated because it is impossible to simultaneously control both the position of the mass-load and the angle between the two linkages. Moreover, the lengths of the two linkages is an internal degree of freedom that is not directly controlled by the hands, instead it is also determined by the position of the virtual mass-load as it interacts with the external force field.

The dynamics of the VUBM is characterized by the following equation, in which [*x,y*] are the output variables, and [*x_1_,y_1_*] (position of one handle) and [*x_2_,y_2_*] (position of the other handle) are the input variables:
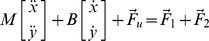
(3)


VUBM is simulated by integrating the equation above over time and transmitting to the two robots the corresponding force vectors 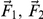.

The overall stiffness matrix of VUBM, which characterizes the interaction between the virtual mass and the force field, is defined by
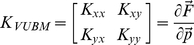
(4)where *F* is the total force applied to the virtual mass and *p* = [*x*,*y*] is its position in the workspace. By computing the partial derivatives, it is possible to obtain the explicit dependence of the four elements of the matrix from the coefficients of elasticity (*K*_s_, *ρ*_s_) and the positions of the two hands with respect to the virtual load:
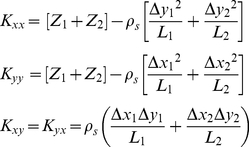
(5)where 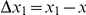; 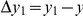; 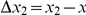; 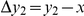. The element of the stiffness matrix that is most relevant from the point of view of stability is *K_xx_*, aligned along the unstable manifold. Supposing that both springs are stretched by an equal amount *L* (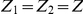), *K_xx_* has a range of possible values between a maximum of 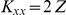, when both springs are aligned with the *x*-axis, and a minimum of 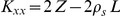, when both springs are aligned with the *y*-axis. If *L* = 0, i.e. if the two hand positions coincide, the stiffness ellipse become a circle of radius 2 *K*_s_.

**Figure 2:**
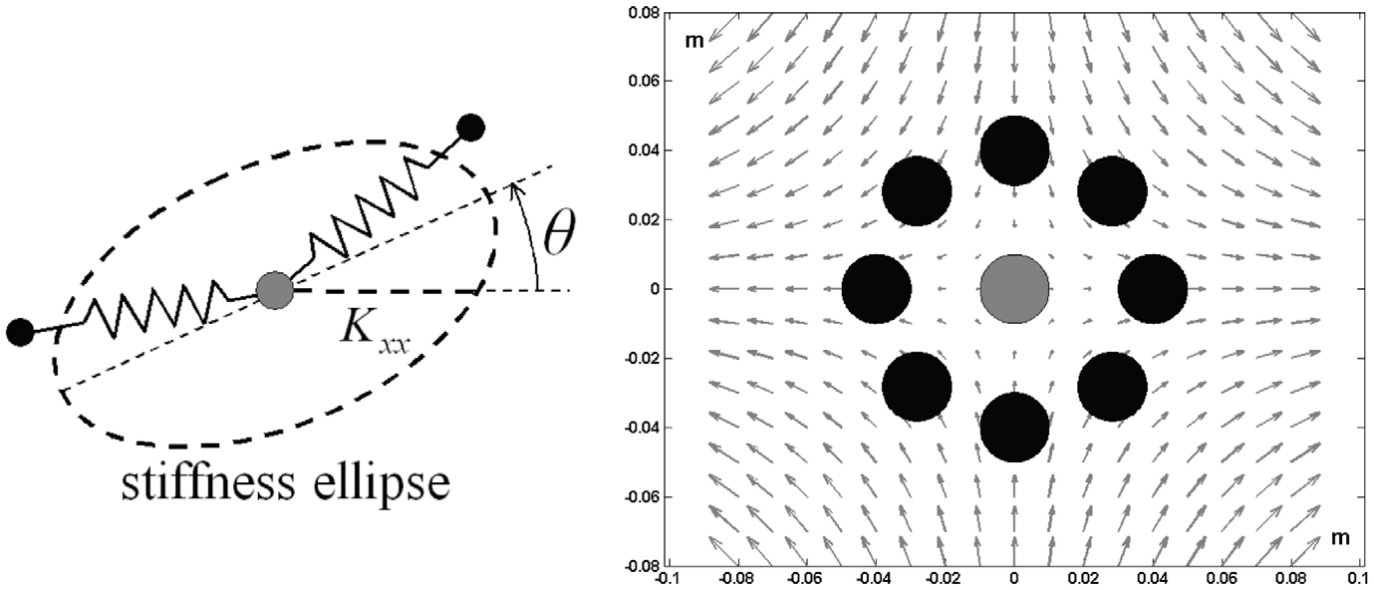
Stiffness parameters and unstable force field. Left panel: Stiffness ellipse, with the characteristic indices: *SSI* (Stiffness Size Index: *K_xx_/K_u_*) and *SOI* (Stiffness Orientation Index* = |cos θ|*). Right panel: Target distribution (circles with 2 cm diameters). The intensity of the force field in the middle of the central target is zero while at the peripheral targets is 23.7 N. At the margin of the workspace it reaches 50 N.

The orientation and size of the stiffness ellipse can be computed from the eigenvalues and eigenvectors of the stiffness matrix. Even though the VUBM is underactuated, subjects can control size and orientation of the ellipse: the major axis is approximately aligned with the line that connects the two handles and the size of the ellipse monotonically grows with the degree of stretch of the two linkages. As shown in Figure 2 (left panel) the stiffness ellipse is characterized by two indexes, which will be used in the analysis of the results:

1. Stiffness Size Index: 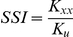

2. Stiffness Orietation Index: 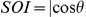

*SSI*>1 suggests that a subject is using the SSS strategy, whereas *SSI*<1 is an indicator of PSS strategy. Moreover, an *SOI = 1* indicates that the major axis of the stiffness ellipse is along the unstable x-axis and while an *SOI = 0* indicates that the axis is oriented along the stable y-axis.

### Systems Parameters

We chose the systems parameters of the VUBM such that the stabilization task was challenging but doable with both types of stabilization strategies: *M* = 15 kg, *B* = 132 N/m/s, *K_u_* = 592 N/m, *K_s_* = *K_u_/4* = 148 N/m, and *ρ_s_* = 1480 N/m^2^. These parameters ensured “well-behaved” dynamics along the stable manifold 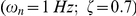
and a falling time constant (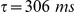) along the unstable manifold that was long enough to allow for feedback stabilization.

The stiffness strategy was not possible near the center of the force field when the two hands were kept close to each other. In fact, when the position of the two hands coincided it yielded an overall stiffness that was isotropic in shape with a magnitude that was equal to only half the critical stiffness (*K_xx_ = K_u_/2* or *SSI* = 0.5). The total stiffness of the VUBM could be increased by separating the hands and stretching the two linkages. Marginal stability (i.e. *K_xx_ = K_u_*) was achieved when the linkages were sufficiently stretched. For example, if the virtual mass was in the center of the field, marginal stability could be obtained by aligning the two linkages on the *x*-axis and stretching each of them by *L = *5 cm. At this length, the force transmitted by each hand to the load is *F = *11.1 N. However, this is not the only solution, although it implies the lowest possible effort. The solution with the highest effort occurs when the two linkages are aligned along the *y*-axis; in this case it is necessary to double the amount of stretch (*L = *10 cm) on each linkage, which requires more than double the effort (*F = *29.6 N).

Summing up, the parameters of the unstable dynamics and those of the control linkages were chosen in such a way that both stabilization strategies (SSS and PSS) were possible: 1) PSS because the falling time constant was sufficiently long and 2) SSS because the critical stiffness could be achieved, although at the expense of a larger but manageable effort. One may also wonder if the endpoint stiffness of the two hands was sufficient to stabilise the load with the SSS strategy. Franklin and Milner [25] suggested an upper range of 600 N/m for the stiffness of the hand. Since our setup is bimanual the overall stiffness is the cumulative stiffness of both linkages. To achieve the SSS strategy the *K_xx_* must be greater than 592 N/m. This suggests that the stiffness of each hand must at least be 296 N/m, which is nearly half the upper threshold suggested by Franklin and Milner [25].

### Task and protocol

The subjects sat in a chair, with their trunk restrained by means of a seat belt and their sternum aligned with the midline of the bimanual robot. At the start of the experiment the two hands were positioned at the center of the force field, which resulted in unloaded elastic linkages (*L*_1_ = *L*_2_ = 0). Then the force field was switched on for the remainder of the experiment. Subjects were asked to stabilize the mass within circular targets that had 2 cm diameters (Figure 2, right panel) for an uninterrupted period of 4 s. The positions of the targets, the mass-load, and the two handles were represented as small circles on a large computer screen, placed vertically in front of the subjects. The orientation of the two elastic linkages were also displayed by means of two segments that joined the load-mass to the two handles.

Nine target were used: a central target, located at the origin of the force field, and eight peripheral targets, uniformly arranged on a circle with a 8 cm diameter. As soon as a target was visualized on the computer screen, the task was to bring the virtual mass inside the target and keep it there as precisely as possible, until the target was switched off and another one was activated. Subjects proceeded on to the next trial after they stabilized the mass inside the current target for an uninterrupted period of 4 s. Any momentary exit, before the prescribed deadline, caused a counter to reset. The prescribed 4 s duration for maintaining equilibrium inside the target area stemmed from a trade-off between two requirements: 1) to avoid too long experimental sessions and 2) to ensure that stabilization was an active process, not a random, momentary event.

The experimental paradigm was organized into *trials*, *target-sets*, and *phases*. Subjects knew they had completed the trial successfully when the old target was switched off and a new one appeared. A *target-set* consisted of 16 trials. In every other trial, subjects were asked to stabilize on a target located at the center of the workspace. Stabilization at the central target was followed by stabilization at one of the randomly selected peripheral targets. We focused our analysis on the central target because it was the most relevant in terms of stabilization. At the center of the workspace, the intensity of the force field was zero and thus the VUBM assumed a straight configuration, with the two elastic linkages equally stretched in opposite directions. The subject were free to choose the orientation and the elongation of the VUBM. Both these aspects determined the orientation and size of the stiffness ellipse. The fact that subjects were fully in control of the magnitude of the stiffness ellipse allowed for a clear-cut distinction between the PSS and the SSS strategy.

In contrast, at the peripheral targets subjects had to compensate for a bias force due to the 4 cm distance of these targets away from the center of the workspace. At each peripheral target, the bias force had a magnitude of 23.7 N but its direction was dependent on the position of the target. Even if the two hands were kept close together, the bias force elongated both linkages thereby increasing the overall stiffness of the VUBM and biasing the strategy selection in the direction of the SSS. The task was designed for examining stability mechanisms in the center of the force field and the shifts to peripheral target areas had the purpose of evaluating the robustness of the mechanism by introducing movement transients characterized by equilibrium breaking and recovery.

The entire protocol consisted of two phases:

*the familiarization phase*: which included the first three target sets at the beginning of each experiment. This phase was used to introduce naïve subjects to the experimental apparatus and to the task protocol. During this phase, the unstable force field along the x-axis was absent and the convergent field along the y-axis however was present.
*the adaptation phase*: which included the last six target-sets of the experiment. During this phase the unstable component of the force field along the *x*-axis was active. All together, in the adaptation phase each subject stabilized on the central target for 48 different trials.

For most subjects, the familiarization phase typically lasted less than five minutes. The adaptation phase was much longer and variable from subject to subject. In order to minimize fatigue, subjects were given a two-minutes rest period after every 24 trials. Prior to the onset of the unstable field, in the adaptation phase, subjects were asked to bring their hands close to the mass. In this configuration, the length of the linkages was close to zero; this ensured that all subjects initiated the adaptation phase with a state of maximum instability. The last two target sets also included *catch trials*, in which the unstable force field was unexpectedly removed for randomly selected targets.

### Data Analysis

The trajectories of the two hands and the virtual mass, the position of the target, and a Boolean flag indicating a catch trial were collected and stored at a frequency of 100 Hz. The time intervals corresponding to the 4 s stabilization periods at the central target was identified offline. During each interval, the mean value of the first five indicators listed below were computed. The final indicator was calculated based on the entire trial period, which included transition to the target and stabilization at the target.

1. Siffness Size Index (*SSI*)
2. Stiffness Orientation Index (*SOI*)
3. Bimanual Separation Index (*BSI*), defined as the distance between the two hands.
4. Correction Burst Frequency (*BF*): it is an indirect measure of the average number of force impulses used to stabilize the mass. It is estimated by counting the total number of peaks in the speed profile of each hand and normalizing by the duration of the target set. A peak is defined as a local maximum that is at least 20% higher than the average speed exhibited during the trial. Although arbitrary, the 20% threshold is not critical because the estimated burst frequency is not altered significantly at a threshold of 15% or 25%. Each local maximum was evaluated within a 100 ms interval. The reported burst frequency is the average across both hands.
5. Effort index (*E*), defined as the sum of the magnitudes of the forces delivered by the two hands, divided by 2.
6. Cumulative Effort *(CE)*,which was introduced in order to quantify the effort applied during the entire trial time. It is defined as the integral of force over the entire trial, which includes both the time interval required to transition from a peripheral target to the central target and the 4 s stabilization period at the central target: 
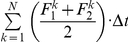
, where N is the number of frames in each trial, *Δt* is equal to the sampling time (0.01 s), 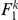 and 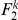 are the magnitude of the force applied by the left and right hands during time step *k*.

Subjects were classified according to their average *SSI* index during the last two target sets. During this period, all subjects were sufficiently familiar with the unstable force field and had adopted a consistent strategy for stabilization. Subjects were placed in the SSS group if their average *SSI*≥1 or into the PSS group if their average *SSI*<1. The data from the two groups during the last two target sets were compared using an ANCOVA, with subjects as a random variable (*p≤0.05*). All mean values are reported with the corresponding ±1standard error.

## Results

The bimanual reaching task was quickly mastered during the familiarization phase. In fact, all subjects applied the PSS strategy with both hands in close proximity, with an average *BSI* of 5.0±3.2 cm. This suggests that in the absence of an unstable field, the springs were virtually unloaded. Moreover, subjects simplified the control problem by reducing the degrees of freedom such that both hands translated as a single unit with the mass.

### Initial behavior in the adaptation phase

**Figure 3:**
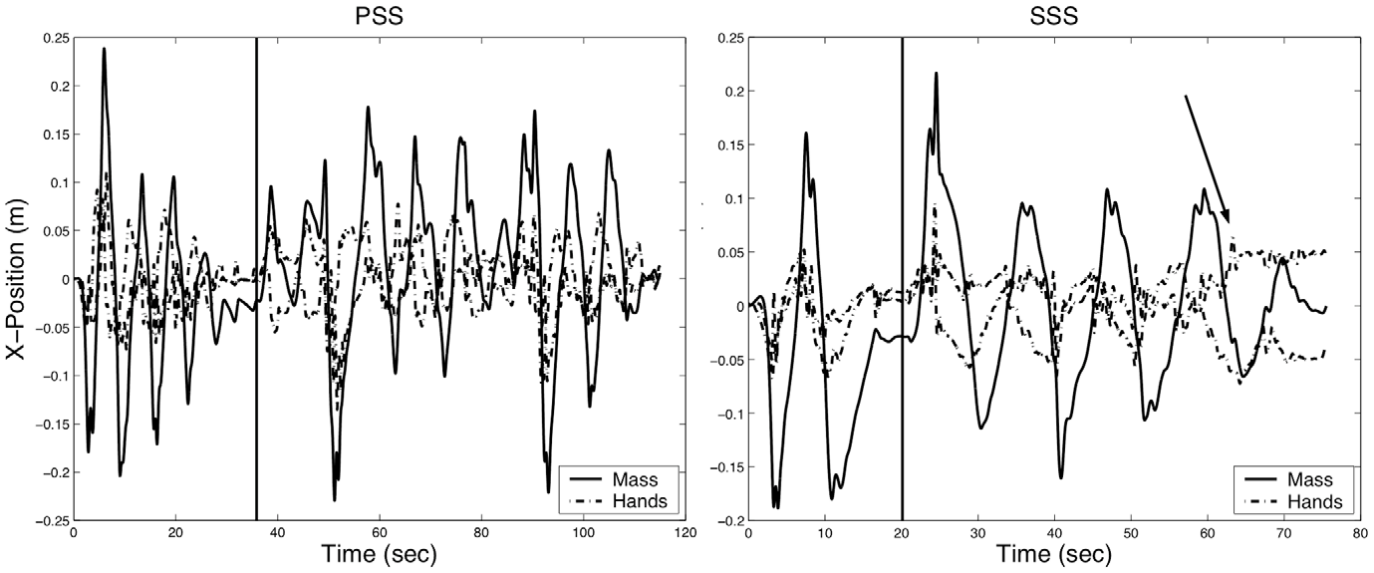
Hand coordination along the unstable axis. Evolution of the mediolateral component (x-axis) of the movement of the two hands and the virtual mass during the first two trials in the adaptation phase for two representative subjects: S6 (left panel) who was classified as a PSS user and S10 (right panel) who was classified as a SSS user. Note that at start of the adaptation phase both the hands and the virtual mass were located in the origin of the force field. The vertical line indicates the end of the first trial at a peripheral target, i.e. the time required to stabilize the mass for an uninterrupted period of 4 s. The interval following the vertical line plots the transition and the stabilization at the central target. The arrow in the right panel highlights the point at which the SSS subject separated the two hands along the unstable direction.

Initial exposure to the force field at the onset of the adaptation phase was characterized by large oscillations of the virtual mass. In fact, the range of movements exhibited by the mass were much greater than the range of movements experienced by either hand (Figure 3). This suggests that the oscillations were a consequence of the unstable force field and that bounded stability was achieved through passive stretch on the elastic linkages of the VUBM rather than with an active control strategy. These large movements helped expose subjects to a large portion of the workspace and to the range of unstable forces that were associated with the task. Moreover, the small range of motion of the hands suggests that the hands operate as stiff position controllers, with stiffness greater than the instability coefficient of the field. This applies not only to the initial behavior when the subjects are still unable to stabilize the load-mass, but also to the later phases when the two stabilization strategies emerged (see below).

### Emergence of two different stabilization strategies

Within one to two minutes of exposure to the unstable task, organized control patterns began to emerge. All subjects were able to reduce the oscillations of the mass such that it remained within the target region at the end of each trial. However, not all the subjects exhibited the same stabilization strategy. Some subjects applied a bimanual coordination pattern similar to the one seen during the familiarization phase, i.e. the hands were kept close to each other and the VUMB operated as a single elastic element. This strategy required an intermittent sequence of force bursts (i.e. the PSS strategy) in order to constrain the movement of the mass to remain inside the target area. In contrast, other subjects found that by separating the hands and stretching the springs along the unstable x-axis they could increase the stiffness of the VUBM and thereby reduce movement of the mass due to the unstable force field (i.e. the SSS strategy). The SSS strategy resulted in *asymptotic stability*, with little need for active control once sufficient stiffness was achieved. In contrast, the PSS strategy led to a weaker form of *bounded stability*, which relied on a persistent sequence of active stabilization commands.

### Characterization of the two stabilization strategies

**Figure 4:**
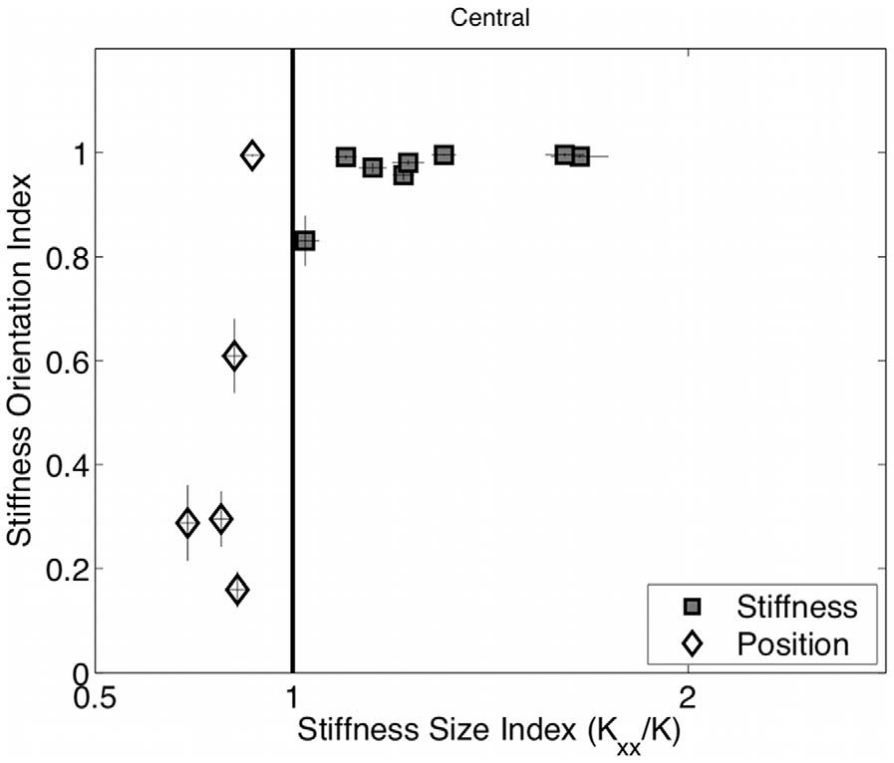
Stiffness magnitude versus orientation. *SOI* vs. *SSI* at the central target during the stabilization interval in the last two target sets of the adaptation phase. The average values for each subject with standard error bars are shown.

During the last two target sets, the performance of all the subjects could be characterized by repeatable stabilization patterns. At this point, eight of the thirteen subjects applied values of the *SSI* that were consistently greater than one and thus were classified as the SSS users. As show in Figure 4, these subjects applied stiffness ellipses that were well aligned with the unstable manifold (the *x*-axis) with *SOI* values that were close to one. The remaining five subjects demonstrated *SSI* that were less than one and thus were classified as PSS users. Unlike the SSS group, these subjects showed no clear preference for the orientation of the stiffness ellipse. This difference can be easily explained by examining the *BSI* index between the two groups (on average PSS: 10.2±4.04 cm vs. SSS: 17.31±5.54 cm, *p = 0.01*), see also Table 3. As can be seen in Eq. 5, the smaller the separation between the two hands at the central target, the smaller the difference between *K*_xx_ and *K*_yy_. As a consequence, the length of the stiffness ellipses along the x-axis was smaller for PSS users than for SSS users.

**Table 3:** Performance Indicators.

Sub	*SSI*	*SOI*	*BSI*	*BF*	*E*	*CE*	*STR*
S1	1.28±0.11	0.96±0.04	16.29±2.27	2.46±0.49	22.10±4.49	151.43±47.35	SSS
S2	1.76±0.29	0.99±0.01	25.16±5.81	1.76±0.53	43.24±14.75	236.55±81.34	SSS
S3	0.73±0.09	0.29±0.29	7.96±2.63	2.63±0.29	8.63±3.60	141.68±102.36	PSS
S4	1.68±0.20	1.00±0.01	23.84±3.85	2.12±0.60	39.21±9.61	219.25±53.91	SSS
S5	0.89±0.11	0.99±0.01	7.99±2.21	2.30±0.19	8.67±2.90	190.78±141.63	PSS
S6	0.85±0.14	0.61±0.29	9.76±3.66	1.97±0.36	11.42±5.90	344.55±309.39	PSS
S7	0.82±0.10	0.30±0.21	11.31±3.96	2.62±0.43	13.81±6.36	211.95±132.17	PSS
S8	1.13±0.11	0.99±0.01	12.77±2.14	3.06±0.24	15.86±3.39	197.65±76.05	SSS
S9	1.38±0.12	1.00±0.01	17.73±2.51	2.11±0.49	25.01±5.04	136.92±26.04	SSS
S10	1.03±0.14	0.83±0.19	12.20±2.44	2.81±0.37	14.88±4.00	168.27±106.41	SSS
S11	0.86±0.11	0.16±0.14	13.84±4.32	2.86±0.19	18.07±7.49	214.44±148.64	PSS
S12	1.29±0.16	0.98±0.02	16.10±3.08	2.49±0.37	21.93±5.89	160.91±47.75	SSS
S13	1.20±0.14	0.97±0.04	14.40±2.67	2.57±0.41	18.64±4.81	119.67±32.59	SSS

Performance indicators at the end of the adaptation phase (last two target-sets) for the stabilization in the central target area. ***SSI*** (Stiffness Size Index): *K_xx_/K_u_*. ***SOI*** (Stiffness Orientation Index): the orientation of the major axis of the stiffness ellipse. ***BSI*** (Bimanual Separation Index): average distance between the two hands [cm]. ***BF*** (Burst Frequency): [impulses/s]. ***E*** (Effort): [N]. ***CE*** (Cumulative Effort): [Ns]. ***STR*** (Stabilization strategy label): SSS/PSS (subjects were classified as users of the SSS or PSS strategy, based on their average behavior during the last two target sets at the central target.

**Figure 5:**
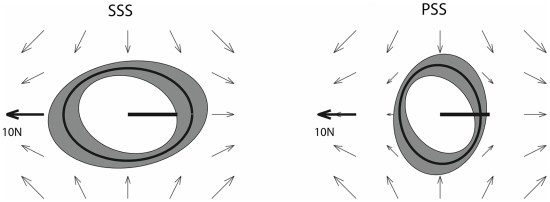
Stiffness ellipse for both groups. Mean stiffness ellipses calculated for all subjects in the SSS and the PSS group during the two final target sets at the central target area. The ellipses are superimposed on a schematic of the unstable force field. The arrow to the left of each panel is the scale factor corresponding to the external force field. The bold horizontal line is the scale factor for the stiffness ellipses; its length corresponds to a stiffness value of 592 N/m, which is equal to the stiffness coefficient of the unstable force field *K_u_*.

The average stiffness ellipse at the central target in the two final target-sets for the subjects in the PSS and SSS group is shown in Figure 5. The SSS users have elongated ellipses, with the major axis aligned along the unstable manifold and an amplitude that is larger than the critical stiffness (*K*_u_ = 592 N/m). In contrast, the magnitude of the stiffness ellipses of PSS is smaller than the critical stiffness and its shape is closer to round.

**Figure 6:**
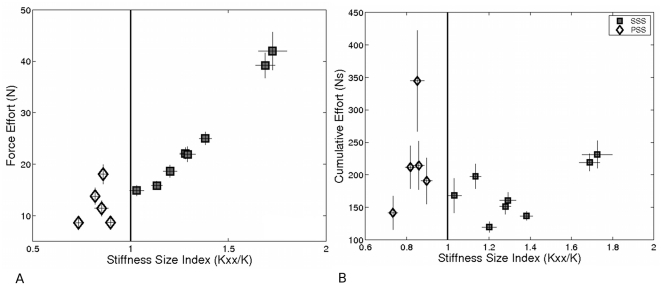
Stabilization strategy versus effort. A) *E* vs. *SSI* and B) *CE* vs. *SSI* at the central target during the last two target sets of the adaptation phase. The average values for each subject with standard error bars are shown on both graphs.

Figure 6A shows the relationship between the average effort applied during the stabilization period and the stabilization strategy implemented by each subject. During the 4 s stabilization phase, the force efforts were significantly larger (*p = 0.02*) for SSS subjects as compared to their PSS counterparts. In fact, on average the SSS group applied twice as much effort (SSS: 25.1±12.3 N vs. PSS: 12.1±6.45 N) as the PSS group. When applying the SSS, effort was used to *load* the virtual springs in order to achieve asymptotic stability, whereas within the PSS group, effort was used to transmit small force bursts to prevent the mass from leaving the target region. The PSS group also took significantly longer to reach the target than the SSS group (19.3±2.7 s versus 5.2±2.1 s; *p = 0.002*). This is a mechanical consequence of the fact that high-stiffness strategy implies a larger frequency bandwidth than the low-stiffness strategy. With the high stiffness strategy subjects continuously applied large forces but stabilized quickly, whereas with the low stiffness strategy subjects applied force bursts but required longer to stabilize. Thus, it is not surprising that the cumulative effort (Figure 6B), which takes into account the trial time, is not significantly different between the two groups *(p = 0.17)*.

**Figure 7:**
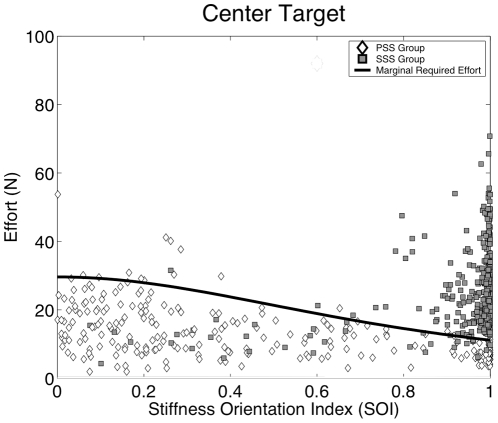
Effort and stiffness orientation affect choice of stabilization strategy. Plot of the relationship between *E* and *SOI* during the stabilization period at the central target area. Data from every trial in the adaptation phase (48×13 = 624 trials) is presented. Gray squares represent SSS subjects while white diamonds represent PSS subjects. The black curve plots the amount of force effort required to reach marginal stability, i.e. the condition in which *K_xx_ = K_u_*.

Further insight into the control strategy can be gained by considering the effort necessary to reach *marginal asymptotic stability* as a function of the *SOI*. Marginal asymptotic stability occurs when the stiffness of the virtual manipulandum along the direction of the unstable manifold equals the coefficient of instability of the force field: *K*_xx_ = *K*_u_. Figure 7 shows this curve, together with a plot of each trial in the adaptation phase. The curve of marginal stability is obtained by considering the first element of Eq. 5 (*K*_xx_) and equating it to *K*_u_. In order to reach marginal stability at the central target, the force applied by one spring must be equal and opposite in direction to the force applied by the other spring. This can only be achieved when the two opposing springs are equal in length: *L*_1_ = *L*_2_ = *L*, Δ*x*_1_ = Δ*x*_2_ = *L* cosθ. Based on the definition of the *SOI* = |cos(θ)|, and the effort term *E* = *K*_u_/4 *L*+ρ_s_ *L* ^2^ the analytic formula for the curve of marginal stability is as follows:
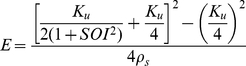
(6)


As highlighted by the figure, the effort necessary to reach marginal asymptotic stability is more than twice for *SOI* = 0 than for *SOI* = 1. The SSS users are clustered on the right-hand side of the plot, avoiding the additional effort they would have incurred had they adopted a non-optimal stiffness orientation. The PSS users, on the other hand, hover consistently below the curve, with a significant lower level of effort than the SSS users.

In order to determine whether physical features indicative of ‘arm strength’ played a role in the choice of a control strategy, the weight, the body mass index, and the grip force were compared between the two groups. However, the results of these various parameters were mixed. The SSS subjects had significantly greater weight than the PSS subjects (70.9 kg vs. 59.2 kg; *p = 0.03*), the body-mass index (SSS: 22.96 kg/m2 vs. PSS: 20.79 kg/m2) was only marginally significant (*p = 0.06*), and the grip force (SSS: 411 N vs. PSS: 347 N) was not significantly different (*p = 0.68*) between the two groups. From these results it remains unclear whether adopting one strategy over another was based on physical factors.

### Catch trials

**Figure 8:**
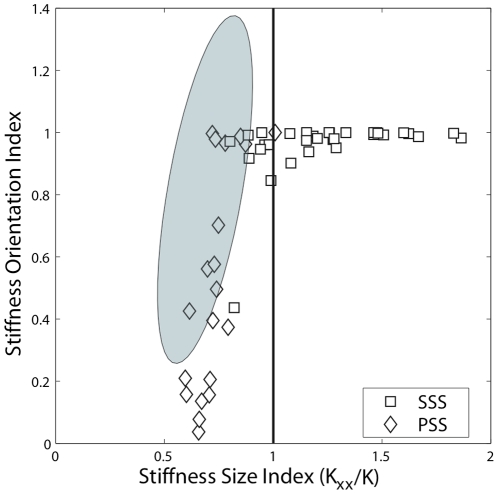
Stiffness characteristics remain constant during the catch trials. *SSI* vs. *SOI* during the catch trials (4 trials for each subject during the last two target sets) in the central target. The squares and the diamonds represent trials from subjects who were classified as SSS or PSS users, respectively. The shaded area spans the trials from the familiarization phase (within two standard deviations).

The last two target-sets also included four catch trials during which the unstable component of the force field was unexpectedly removed. Figure 8 illustrates the relationship between the *SSI* and the *SOI* during the catch trials for the entire subject population. What is relevant is that a sudden and unexpected elimination of the source of instability did not alter the pattern of control for either the SSS or the PSS group. In fact, the subjects retained the strategy they had adopted when the divergent component of the field was active. In particular, during the catch trials SSS subjects maintained *SSI>1* similar to that applied during the adaptation phase, while PSS subjects used *SSI* values that were comparable to those of the familiarization phase (shaded area in Figure 8).

### Stabilization bursts

When the subjects stabilized the virtual mass within the target area, small but persistent oscillations were observed, similar to the sway movements observed during quiet, upright standing. Interestingly enough, the frequency of correction bursts were not significantly different between the two groups (*p* = 0.9 with an average bursting frequency of 2.6 bursts/sec). However, the role of such correction bursts appears to be different. In the case of the SSS users, the stiffness of the virtual manipulandum is beyond the critical stiffness and thus there is no need, in principle, for a persistent train of stabilization bursts. Moreover, these peaks are too small to compensate for the external field. Instead they may have been used to counter the internal noise from high muscle activity [26] that was required to stretch and stiffen the linkages of the VBM. In the case of the PSS users, the speed peaks are much higher and subsequent peaks are more distinct. Since the effort for this group during stabilization period was relatively low, the control bursts were likely used to compensate for the external force field rather than internal noise from the muscles.

### Robustness in the choice of a stabilization strategy

Subjects were exposed to a wide range of forces and stiffness values during the initial exploratory phase at the onset of the unstable force field, and when switching between the central and the peripheral targets. For example, when switching from the central to the targets located along the unstable x-axis, the additional task of compensating for the bias force of 23.7 N pushed the PSS users to a stiffness beyond the critical value, thus turning them, temporarily, into SSS users. Nevertheless all of them went back to the low-stiffness stabilization regime when returning to the central area. This suggests that the strategy implemented at the central target for stabilization was robust.

### Summary of the difference between the two stabilization strategies

The main features that arise from stabilizing an unstable load by means of a virtual manipulandum with an adjustable impedance can be summarized as follows:

1. Two groups consisting of approximately equal number of subjects adopted the two theoretically possible stabilization strategies.
2. The choice was made early on in the learning process and maintained throughout the experiments in spite of catch trials and frequent destabilization periods associated with target switching.
3. The effort used for stabilization by the SSS subjects was nearly twice the effort used by PSS subjects, however the cumulative effort was not significantly different between the two groups.
4. Unlike the PSS users, the stiffness ellipse for the SSS subjects were elongated with the major axis oriented along the unstable *x*-axis.
5. In the case of SSS users we can assume that the predominant part of the control is open-loop. Once the critical level of stiffness had been achieved by separating the hands and loading the springs little active control was necessary. Alternatively, PSS users relied heavily on trains of stabilization bursts in a closed-loop mode of control, which monitors the system's oscillations. In other words, SSS users primarily controlled the stiffness of the virtual manipulandum, whereas PSS users primarily controlled the force impulses transmitted to the manipulandum.
6. The two different stabilization strategies suggest that a single criterion is not being optimized; instead the skills acquired by the two groups appear to be *locally* optimal. For example, the ellipse orientation used by the SSS group is optimal for a stabilization strategy characterized by stiffness. In contrast, the PSS subjects tend to minimize the energy stored in the virtual manipulandum by keeping the hands close together.

## Discussion

Many studies have investigated different control strategies ranging from stiffness strategy to an intermittent mode of feedback control. However, unlike previous studies, which have mainly focused on the mechanisms underlying these strategies, this experiment was designed to examine how different strategies were chosen in a task that caters to two different solutions for stabilization. The first solution is based on a high stiffness strategy (SSS), while the second solution is based on a low-stiffness position feedback control (PSS). Stiffness can also be considered a positional feedback mechanism but since the stiffness is determined by the material properties of the muscles and/or the manipulandum, the feedback is “implicit”. A stiffness mechanism essentially “overpowers” the dynamic phenomenon generating the instability by means of a stronger convergent field. If the convergent field is set such that it is beyond a critical value, a specific regulation for preserving stability or for responding to perturbations becomes unnecessary. Moreover, feedback associated with stiffness is nearly instantaneous, aside for the quick transients related to the properties of the material. However, the SSS mechanism is not trivial; practice is required before one can learn to adjust the stiffness ellipse to match the instability in the environment in an optimal way. In contrast, low-stiffness PSS requires “explicit” feedback from sensory receptors on the state of the body in relation to the environment. For positional feedback control, corrective stabilization commands must be generated persistently, either in a continuous manner as smoothly varying signals, or in an intermittent manner as a sequence of correction bursts. In both cases the feedback signals are delayed, which introduces an additional source of instability. Consequently, stabilization via a PSS is intrinsically band-limited, typically on the order of 1–2 Hz. In contrast, SSS does not have such strong limitation because it is similar to a “preflex”, in the sense defined by [27]. In summary, the SSS is a high-bandwidth, high-effort mechanism whereas the PSS is a low-bandwidth, low-effort mechanism.

In this study, a haptic bimanual robot was used to simulate a virtual bimanual device, with two non-linear elastic linkages. During the experimental task, human subjects learned to stabilize the end-effector of the device while it was under the action of an unstable, saddle type force field. This field is characterized by a divergent component along the mediolateral direction and a convergent component along the anterioposterior direction. Moreover, the linkages had a quadratic length-tension curve such that the stiffness of each linkage increased with its length. Thus, by sufficiently stretching the virtual manipulandum subjects could alter the stiffness of the virtual manipulandum in order to reach asymptotic stability. Alternatively, they could transmit force impulses via the linkages to correct for movement errors of the end-effector, thereby achieving bounded stability within the target region.

The experiment was designed to promote exposure to the mechanisms underlying the PSS and the SSS strategies. At the onset of the unstable force field, both linkages were unloaded and the stiffness of the virtual manipulandum was at its lowest possible value, i.e. half the coefficient *K_u_* of the unstable field, which is equal to an *SSI* of 0.5. Thus, stiffness of the virtual manipulandum was too low to asymptotically stabilize the load. As a consequence, the virtual mass oscillated back and forth along the unstable direction. During this period subjects were exposed to a broad range of forces and stiffness. Stabilization at the peripheral targets also led subjects to adopt a range of stiffness magnitudes and orientations. The peripheral targets were positioned at a distance of 4 cm from the center of the workspace. At these locations, in addition to stabilization, subjects had to compensate for a bias force of 23.7 N. The orientation of the bias force was based on the location of the peripheral targets, which can ultimately result in a variety of stiffness values for a given bimanual coordination strategy. For example, if we consider the bimanual coordination typically used by PSS subjects, i.e. keeping the two hands very close to each other, the forces delivered by each hand will be the same irrespective of the peripheral target (*F_1_* = *F_2_* = 11.85 N). However, the stiffness values will be quite different. For example, the *SSI* is 1.02 for the two targets on the x-axis, 0.89 for the four targets at 45° and 135°, 0.76 for the two targets on the y-axis, and 0.5 for the central target. This suggests that even after learning, the subject experienced a dynamic environment with strongly variable stiffness requirements and thus in principal they could easily switch from one strategy to another if they wished. Despite this fact, subjects adhered to the strategy learned early on in the experiment. Zenzeri et al [28] found that a well-trained subject can easily switch from one strategy to the other. However, the present study was limited to the initial phase of learning for naïve subjects and showed that the choice of the strategy emphasized one aspect or the other of the task. SSS subjects seem to be predisposed toward maintaining stability even at the cost of applying large forces. In fact, they often applied forces that exceeded the effort necessary to achieve marginal stability. The PSS subjects on the other hand were more conservative about applying large forces. They preferred to rely on feedback to generate a well-timed series of movements to correct for deviations of mass from the central target.

It remains unclear whether physical factors such as arm ‘strength’ played a role in strategy selection. Factors such as the average weight were significantly different between the two groups (*p = 0.03*), while other factors such as BMI and grip strength were not (*p = 0.06* and *p = 0.68*, respectively). Nevertheless, even if one group had greater arm ‘strength’ than the other, the data indicates that both groups were strong enough to apply the high stiffness strategy. In fact, the average force in the SSS group was sufficient to execute the high stiffness strategy at stiffness orientations ranging from *SOI* = 0 to *SOI* = 0.34 (i.e. 0°–70° from the x-axis), while the average effort demonstrated by the PSS group (*E* = 13.2 N) was fact sufficient to adopt the SSS strategy if the stiffness orientation was along the unstable x-axis (a minimum force effort of 11.1 N is required to execute the SSS strategy at a *SOI* = 0).

Generally speaking it does not appear that the observed behavior can be explained in terms of global optimization of effort. Instead subjects adopted two distinct strategies that apply effort in different ways. The SSS subjects continuously applied large forces to increase stiffness and rapidly achieved asymptotic stability, while PSS subjects applied force impulses and took longer to stabilize the mass. Interestingly enough, all SSS subjects adopted a stiffness orientation that allowed them to reach marginal stability with the lowest amount of effort. And although these novice subjects applied forces that far exceeded the amount of effort needed to reach marginal stability, we believe that orienting the stiffness ellipse along the unstable x-axis was an initial step towards reaching a more optimal solution, which allows for asymptotic stability with lower effort. In fact, Zenzeri et al [28] showed that with extensive practice the amount of effort applied by subjects gradually decreased. In contrast, the PSS subjects were less stringent about the stiffness orientation than the SSS group, and exhibited no clear preference for the stiffness orientation.

Aside from a recent study by Ganesh et al [22], most studies on modelling the neural control of movement have been formulated in terms of optimising a cost function related to physiological and/or task variables such as motion smoothness [29], joint torque [30], motor noise [26], a combination of error and effort [31], just to name a few. Unlike the current study, the cited investigations were aimed at global optimisation, where subjects were supposed to search for a unique optimal solution to a given task. The issue of suboptimality was limited to address incomplete convergence to the unique optimum [32] rather than a characterization of experimental paradigms with task-relevant multiple optima. In contrast, real life tasks that require skilled control of tools in a variable, partially unknown environment are likely to require the ability to switch from one strategy to another. Moreover, in the course of an action subjects will likely accept suboptimal criteria that are sufficient to satisfy the task requirements. In this sense, the existence of multiple optimas and the ability of the subjects to access them is a key element of skilled behavior.

In summary, this study examined how subjects stabilized an external object in an unstable environment. The experimental task was designed such that stabilization could be achieved using two distinct control strategies, one based on high stiffness and another based on low stiffness positional feedback. The data indicates that as a whole the population applied both strategies, with nearly half the subjects adopting the positional feedback strategy and the remaining adopting the high stiffness strategy. Those who adopted the stiffness strategy applied a large amount of force for a short period of time and were asymptotically stable, while those who adopted the position feedback strategy used less force for a longer period of time and achieved bounded stability.

## References

[1] Asai Y, Tasaka Y, Nomura K, Nomura T, Casadio M (2009). A model of postural control in quiet standing: robust compensation of delay-induced instability using intermittent activation of feedback control.. PLoS One.

[2] Burdet E, Osu R, Franklin DW, Milner TE, Kawato M (2001). The central nervous system stabilizes unstable dynamics by learning optimal impedance.. Nature.

[3] Franklin DW, Osu R, Burdet E, Kawato M, Milner TE (2003). Adaptation to stable and unstable dynamics achieved by combined impedance control and inverse dynamics model.. J Neurophysiol.

[4] Lakie M, Caplan N, Loram ID (2003). Human balancing of an inverted pendulum with a compliant linkage: neural control by anticipatory intermittent bias.. J Physiol.

[5] Loram ID, Kelly SM, Lakie M (2001). Human balancing of an inverted pendulum: is sway size controlled by ankle impedance?. J Physiol.

[6] Milton JG, Cabrera JL, Ohira T (2008). Unstable dynamical systems: Delays, noise and control.. Epl.

[7] Maurer C, Peterka RJ (2005). A new interpretation of spontaneous sway measures based on a simple model of human postural control.. J Neurophysiol.

[8] Peterka RJ (2000). Postural control model interpretation of stabilogram diffusion analysis.. Biol Cybern.

[9] Bottaro A, Yasutake Y, Nomura T, Casadio M, Morasso P (2008). Bounded stability of the quiet standing posture: an intermittent control model.. Hum Mov Sci.

[10] Loram ID, Gollee H, Lakie M, Gawthrop PJ (2011). Human control of an inverted pendulum: is continuous control necessary? Is intermittent control effective? Is intermittent control physiological?. J Physiol.

[11] Loram ID, Maganaris CN, Lakie M (2005). Human postural sway results from frequent, ballistic bias impulses by soleus and gastrocnemius.. J Physiol.

[12] Loram ID, Maganaris CN, Lakie M (2007). The passive, human calf muscles in relation to standing: the short range stiffness lies in the contractile component.. J Physiol.

[13] Hogan N (1984). Adaptive-Control of Mechanical Impedance by Coactivation of Antagonist Muscles.. Ieee Transactions on Automatic Control.

[14] Gomi H, Osu R (1998). Task-dependent viscoelasticity of human multijoint arm and its spatial characteristics for interaction with environments.. J Neurosci.

[15] Darainy M, Towhidkhah F, Ostry DJ (2007). Control of hand impedance under static conditions and during reaching movement.. J Neurophysiol.

[16] Franklin DW, Liaw G, Milner TE, Osu R, Burdet E (2007). Endpoint stiffness of the arm is directionally tuned to instability in the environment.. J Neurosci.

[17] Perreault EJ, Kirsch RF, Crago PE (2002). Voluntary control of static endpoint stiffness during force regulation tasks.. J Neurophysiol.

[18] Milner TE (2002). Contribution of geometry and joint stiffness to mechanical stability of the human arm.. Exp Brain Res.

[19] Mussa-Ivaldi FA, Hogan N, Bizzi E (1985). Neural, mechanical, and geometric factors subserving arm posture in humans.. J Neurosci.

[20] Perreault EJ, Chen K, Trumbower RD, Lewis G (2008). Interactions with compliant loads alter stretch reflex gains but not intermuscular coordination.. J Neurophysiol.

[21] Maravita A, Iriki A (2004). Tools for the body (schema).. Trends Cogn Sci.

[22] Ganesh G, Haruno M, Kawato M, Burdet E (2010). Motor memory and local minimization of error and effort, not global optimization, determine motor behavior.. J Neurophysiol.

[23] Oldfield RC (1971). The assessment and analysis of handedness: the Edinburgh inventory.. Neuropsychologia.

[24] Casadio M, Sanguineti V, Morasso PG, Arrichiello V (2006). Braccio di Ferro: a new haptic workstation for neuromotor rehabilitation.. Technol Health Care.

[25] Franklin DW, Milner TE (2003). Adaptive control of stiffness to stabilize hand position with large loads.. Exp Brain Res.

[26] Harris CM, Wolpert DM (1998). Signal-dependent noise determines motor planning.. Nature.

[27] Brown IL (1997). A reductionist approach to creating and using neuromusculoskeletal models. Biomechanics and neural control of movement.

[28] Zenzeri JMP, Saha D (2010). Expert Strategy Switching in the Control of a Bimanual Manipulandum with an Unstable Task..

[29] Flash T, Hogan N (1985). The coordination of arm movements: an experimentally confirmed mathematical model.. J Neurosci.

[30] Uno Y, Kawato M, Suzuki R (1989). Formation and control of optimal trajectory in human multijoint arm movement. Minimum torque-change model.. Biol Cybern.

[31] Todorov E, Jordan MI (2002). Optimal feedback control as a theory of motor coordination.. Nat Neurosci.

[32] Izawa J, Rane T, Donchin O, Shadmehr R (2008). Motor adaptation as a process of reoptimization.. J Neurosci.

